# MiR-371a-5p Positively Associates with Hepatocellular Carcinoma Malignancy but Sensitizes Cancer Cells to Oxaliplatin by Suppressing BECN1-Dependent Autophagy

**DOI:** 10.3390/life12101651

**Published:** 2022-10-20

**Authors:** Zhenbing Lv, Xin Qiu, Pu Jin, Zhaodong Li, Yan Zhang, Lei Lv, Fangzhou Song

**Affiliations:** 1 Molecular Medicine and Cancer Research Center, Chongqing Medical University, Chongqing 400016, China; 2 Department of Gastrointestinal Surgery, Nanchong Central Hospital, Nanchong 637000, China; 3 Department of Pathology, Xindu District People’s Hospital, Chengdu 610500, China; 4 Medical Imaging Department of Chongqing Emergency Medical Center, Chongqing 400016, China

**Keywords:** hepatocellular carcinoma, miR-371a-5p, BECN1, autophagy, oxaliplatin, resistance

## Abstract

Oxaliplatin (OXA)-based chemotherapy demonstrates active efficacy in advanced hepatocellular carcinoma (HCC), while resistance development limits its clinical efficacy. Thus, identifying resistance-related molecules and underlying mechanisms contributes to improving the therapeutic efficacy of HCC patients. MicroRNA-371a-5p (MiR-371a-5p) fulfills an important function in tumor progression. However, little is known about the effect of miR-371a-5p on chemotherapy response. In this study, quantitative real-time polymerase chain reaction, Western blot and immunohistochemistry were used to determine the expression levels of miR-371a-5p, BECN1 and autophagy-related proteins in HCC cells, tissues and serum. The luciferase reporter assay was used to assess the directly suppressive effect of miR-371a-5p on BECN1 mRNA translation. Moreover, gain- and loss-of-function assays and rescue assays were used to evaluate the mediated effect of BECN1-dependent autophagy on the role of miR-371a-5p in the response of HCC cells to OXA. We found that miR-371a-5p was significantly up-regulated in HCC tissues and serum from patients, whereas BECN1 protein was down-regulated in HCC tissues compared to the corresponding controls. We also found that there was a negative correlation between the two molecules in HCC tissues. In addition, we found that miR-371a-5p expression was positively associated with malignant characteristics of HCC and BECN1 protein expression is negatively associated. Contrary to this, we found that miR-371a-5p enhances and BECN1 attenuates the response of HCC cells to OXA. Importantly, the enhanced effect of miR-371a-5p on the response of HCC cells to OXA could be reduced by re-expression of non-targetable BECN1, and then the reduced effect was restored following bafilomycin A treatment. Taken together, we identified a dual role of miR-371a-5p in HCC malignant characteristics and the response of HCC cells to oxaliplatin. Importantly, we reveal that miR-371a-5p enhances oxaliplatin response by target suppression of BECN1-dependent autophagy.

## 1. Introduction

Hepatocellular carcinoma (HCC) is the sixth frequent malignancy and the fourth-leading cancer-related cause of death in the world [1,2], accounting for more than 8% of all cancer-related deaths [3], with an overall 5-year survival of patients less than 12% [4]. Most HCC patients are diagnosed late, when curative resection is impracticable. Therefore, new regimens are urgently needed to treat advanced HCC. The oxaliplatin-based chemotherapy regimen has been shown to be effective on advanced HCC [5,6], while resistance development often results in treatment failure. Therefore, uncovering resistance-related molecules and underlying mechanisms helps to improve the therapeutic efficacy of HCC patients.

MicroRNAs (miRNAs) are a cluster of single-stranded, non-coding RNAs with a length of approximately 22 nucleotides. Mechanistically, miRNAs exert their functions by blocking translation of mRNAs into protein via binding to their 3′-untranslated region (3′-UTR) according to the principle of complementary base pairing [7]. MicroRNA-371a-5p (miR-371a-5p), also known as miR-371-5p, has been reported to be involved in a variety of cancer progressions. In colorectal cancer, we have identified miR-371a-5p as a tumor suppressor, which inhibits epithelial–mesenchymal transition, stem cell characteristics and metastasis by targeting SOX2 [8]. Similarly, miR-371a-5p could lower the abilities of proliferation and migration by suppressing BCL2 in nasopharyngeal carcinoma [9]. On the contrary, miR-371a-5p plays a tumor-promoting role in pancreatic cancer via inhibiting ING1 [10]. In HCC, it facilitates G1/S transition by inhibiting PRPF4B [11] and decreases necroptotic cell death by targeting Casp-8 [12]. A recent study demonstrates that down-regulation of miR-371a-5p leads to the enhanced resistance of colorectal cancer cells to cetuximab [13]. Furthermore, miR-371a-5p has been reported to be a biomarker for diagnosing stage IIIA colon cancer [14] and predicting the metastasis of seminomatous germ cell tumors [15]. These findings imply the potential value of miR-371a-5p in governing chemotherapy response and estimating tumor progression. Nonetheless, the impact of miR-371a-5p on HCC chemotherapy response and tumor progression evaluation remain undisclosed.

Autophagy is an intracellular mechanism which orderly degrades and recycles cellular components and plays a dual part in disease progressions including cancer [16,17]. It has been proposed as a potential mechanism to explain the effect of miRNAs on cancer chemotherapy resistance. miR-145-3p elevates the sensitivity of multiple myeloma to bortezomib via autophagy induction [18], by which miR-519a fulfills the same role in the response of glioblastoma to temozolomide [19]. In contrast, miR-541 and miR-125b enhance the response of HCC to sorafenib [20] and to oxaliplatin [4] by autophagy suppression, respectively. However, few studies have addressed the relationship between miR-371a-5p and autophagy experimentally [21], especially in the response of HCC to oxaliplatin. 

In the present study, we identified miR-371a-5p as an oxaliplatin-sensitive gene. We found that HCC patients with high miR-371a-5p expression responded well to oxaliplatin-based chemotherapy, but had a worse tumor phenotype. Importantly, we revealed that miR-371a-5p enhances the response of HCC cells to oxaliplatin by suppressing the BECN1-dependent autophagy pathway.

## 2. Results

### 2.1. MiR-371a-5p Is Associated with HCC Malignant Characteristics and the Response of Patients to FOLFOX4 Therapy

**Figure 1:**
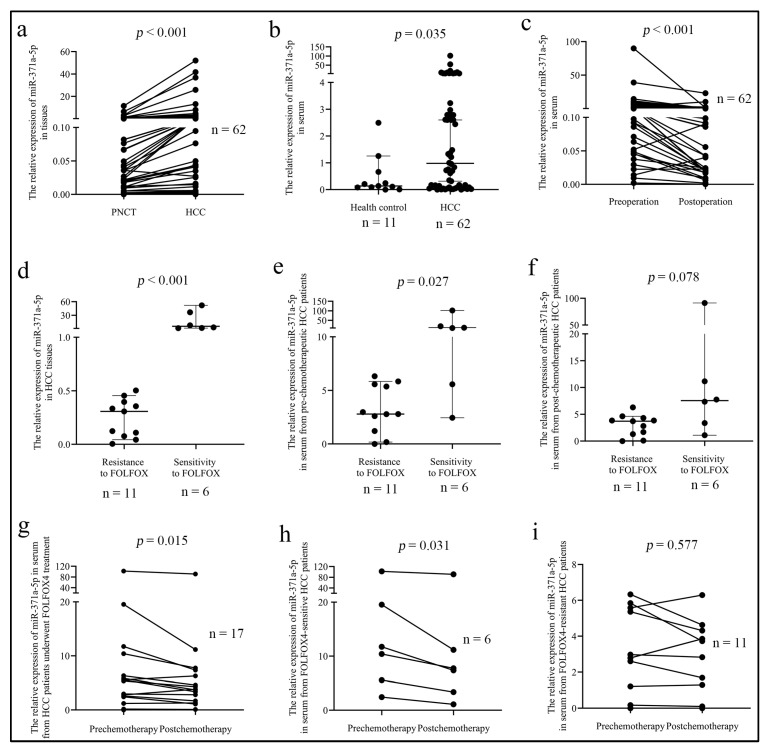
MiR-371a-5p is associated with HCC malignant properties and the response of patients to FOLFOX4 treatment. (**a**) miR-371a-5p expression was detected by QPCR in HCC and the paired adjacent non-cancer tissues (PNCT) (*n* = 62). (**b**) miR-371a-5p expression in serum from 62 prechemotherapeutic HCC patients and 11 health controls was detected by QPCR. (**c**) miR-371a-5p expression in serum from preoperative and postoperative HCC patients was detected by QPCR (*n* = 62). (**d**–**f**) miR-371a-5p expression in HCC tissues (**d**), prechemotherapeutic serum (**e**) and postchemotherapeutic serum (**f**) between 11 FOLFOX4-resistant and 6 FOLFOX4-sensitive patients was detected by QPCR. (**g**–**i**) miR-371a-5p expression in serum from 17 patients underwent FOLFOX4 treatment patients (**g**), 6 FOLFOX4-sensitive HCC patients (**h**) and 11 FOLFOX4-resistant HCC patients (**i**) between pre- and post-chemotherapy was detected by QPCR.

MiR-371a-5p expression was examined by QPCR in 62 cases of fresh HCC tissues, patients’ serum and their counterparts. We found that miR-371a-5p expression was up-regulated in HCC tissues compared to paired adjacent non-cancer liver tissues (PNCT) (Figure 1a). Similarly, miR-371a-5p expression was higher in serum from prechemotherapeutic HCC patients than in serum from health controls (Figure 1b). As shown in Table 1, miR-371a-5p expression in HCC tissues, as well as in serum from prechemotherapeutic HCC patients, was associated with tumor size, tumor differentiation and patient age. Intriguingly, miR-371a-5p expression in HCC tissues from patients with serum AFP > 400 ug/L was higher than that from patients with serum AFP ≤ 400 ug/L, while there was no significant difference in miR-371a-5p expression between the serum of patients from the two group (Table 1). Moreover, we found that miR-371a-5p expression was significantly reduced in serum from postoperative patients compared to in serum from preoperative patients (Figure 1c).

We then investigated the association of miR-371a-5p expression with the response of HCC patients to FOLFOX4 therapy. We found that miR-371a-5p expression was lower in tissue from FOLFOX4-resistant HCC patients than in tissue from FOLFOX4-sensitive HCC patients (Figure 1d). miR-371a-5p expression in serum from prechemotherapeutic HCC patients between the two groups showed the same result (Figure 1e), while there was no statistical significance in miR-371a-5p expression in serum from postchemotherapeutic HCC patients between the two groups (Figure 1f). Moreover, we also found that in HCC patients underwent FOLFOX4 treatment, miR-371a-5p expression was decreased in postchemotherapeutic serum compared to in prechemotherapeutic serum (Figure 1g). The same result was determined in FOLFOX4-sensitive HCC patients (Figure 1h), while there was no difference in serum from FOLFOX4-resistant HCC patients (Figure 1i). These results indicated that HCC patients with high miR-371a-5p expression responded well to FOLFOX4 chemotherapy.

### 2.2. MiR-371a-5p Enhances the Response of HCC Cells to Oxaliplatin In Vitro

**Figure 2:**
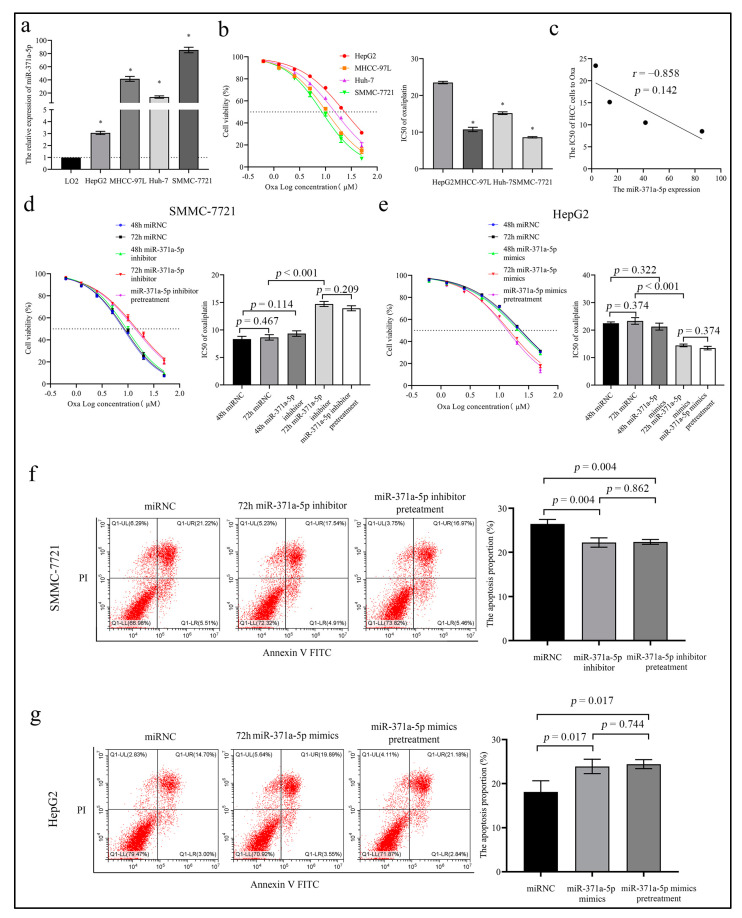
MiR-371a-5p enhances the response of HCC cells to oxaliplatin. (**a**) miR-371a-5p expression in normal liver cell LO2 and four HCC cells was detected by QPCR. The relative expression levels were normalized to LO2 cell (* *p* < 0.05 compared to LO2 cell). (**b**) The cell viability graph and the OXA IC50 statistical histogram in four HCC cells (* *p* < 0.05 compared to HepG2 cell). (**c**) An inverse correlation between miR-371a-5p and the OXA IC50 in the four HCC cells. (**d**,**e**) The cell viability graph and the OXA IC50 statistical histogram in SMMC-7721 cell (**d**) and HepG2 cell (**e**) following the corresponding treatment. (**f**,**g**) The representative FCM images and statistical histogram of OXA-induced apoptosis in SMMC-7721 cell (**f**) and HepG2 cell (**g**) following the corresponding treatment. Three independent experiments were performed.

To investigate the effect of miR-371a-5p on the treatment response, we evaluated the correlation between miR-371a-5p expression and the half inhibitory concentration (IC50) of oxaliplatin (OXA), a main anti-cancer component of the FOLFOX4 regimen in four HCC cells. We found that miR-371a-5p expression was lowest in HepG2 cell and highest in SMMC-7721 cell in the four HCC cells (Figure 2a), while the IC50 of the four cells to OXA showed an opposite trend (Figure 2b). An inverse correlation existed between the two, although the result did not show the statistical difference (Figure 2c). Gain-and loss-of-function assays showed the IC50 of OXA in SMMC-7721 cell was increased in the miR-371a-5p silencing group with the miR-371a-5p inhibitor treatment for 72 h (72 h miR-371a-5p inhibitor) compared to the nonsense control (NC) group (Figure 2d and Supplementary Figure S1a), and the miR-371a-5p over-expression showed an opposite result in HepG2 cell (Figure 2e and Supplementary Figure S1b). Whereas there was no difference in the OXA IC50 between the NC group and the miR-371a-5p silencing/over-expression group with the miR-371a-5p inhibitor/mimics treatment for 48 h (48 h miR-371a-5p inhibitor/mimics), as shown in Figure 2d,e. Noticeably, we found that compared to the 72 h miR-371a-5p inhibitor group, the OXA IC50 in SMMC-7721 cell was not dramatically altered in the miR-371a-5p inhibitor pretreatment group (Figure 2d). The same result was measured in the 72 h miR-371a-5p mimics group in HepG2 cell (Figure 2e). Similarly, flow cytometry (FCM) results of the OXA-induced cytotoxicity showed that the apoptosis proportion of SMMC-7721 cell in the 72 h miR-371a-5p inhibitor group, as well as in the miR-371a-5p inhibitor pretreatment group was reduced compared to the NC group (Figure 2f), and that of HepG2 cell in the 72 h miR-371a-5p mimics group and in the miR-371a-5p mimics pretreatment group showed an opposite result (Figure 2g). These results indicated that miR-371a-5p could enhance the sensitivity of HCC cells to OXA in vitro.

Moreover, we examined the impact of miR-371a-5p on the apoptosis of HCC cells. We found that there was no difference in apoptosis between the miR-371a-5p silencing group and NC group, and between the miR-371a-5p over-expression group and NC group (Supplementary Figure S1c,d).

### 2.3. MiR-371a-5p Represses HCC Cell Autophagy by Target Suppression of BECN1

**Figure 3:**
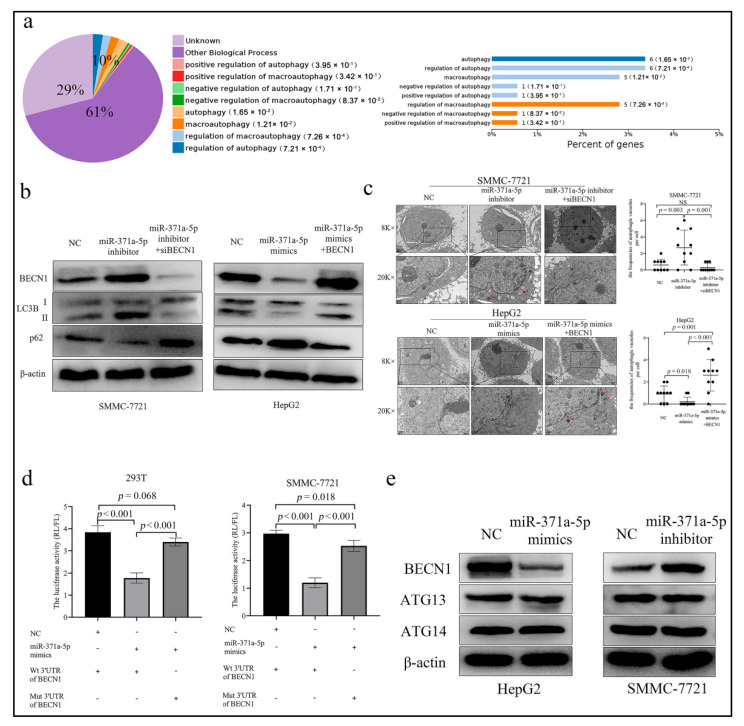
MiR-371a-5p represses HCC cell autophagy by target suppression of BECN1. (**a**) The functional enrichment of miR-371a-5p target genes by OMICSBEAN. (**b**) The protein expression of BECN1, LC3 I/II and p62 was detected by WB in SMMC-7721 cell and HepG2 cell following the corresponding treatment. (**c**) The autophagic vacuoles were detected by TEM in SMMC-7721 cell and HepG2 cell following the corresponding treatment. The representative images were shown and the autophagic vacuoles were marked with red arrows. The frequencies of autophagic vacuoles per cell were calculated by averaging the frequencies of autophagic vacuoles in 10 cells. (**d**) The luciferase activities of Wt and Mut pmirGLO-3′UTRs of BECN1 mRNA in 293T and SMMC-7721 cells following the miR-371a-5p mimics treatment ((Wt wild-type, Mut mutant-type, RL Ranilla luciferase, FL Firefly luciferase). (**e**) The expression of BECN1, ATG13 and ATG14 was determined by WB in HCC cells following miR-371a-5p inhibitor or mimics treatment. Three independent experiments were performed.

Given that miRNAs come into play depending on their target genes, we analyzed potential functions of miR-371a-5p target genes by the on-line software OMICSBEAN (http://www.omicsbean.cn/, accessed on 5 March 2019). The results showed that the functions were implicated in the regulation of biological processes, cell components and molecular functions (Supplementary Figure S2a). Of them, approximately 10% targets were implicated in the regulation of autophagy and macro-autophagy (Figure 3a). To verify the bioinformatic results, we investigated the effects of miR-371a-5p on autophagy in HCC cells. WB results showed that the miR-371a-5p silencing increased the ratio of LC3 Ⅱ/Ⅰ and reduced the SQSTM1/p62 expression in SMMC-7721 cell, while the miR-371a-5p over-expression exhibited an opposite result in HepG2 cell (Figure 3b). Similarly, the results of TEM showed that the miR-371a-5p silencing increased the frequencies of autophagic vacuoles in SMMC-7721 cell, while the miR-371a-5p over-expression decreased the frequencies of autophagic vacuoles in HepG2 cell (Figure 3c).

We sought to explore the mediator responsible for miR-371a-5p-related autophagy in HCC cells. We screened the autophagy-related targets of miR-371a-5p in TargetScan Human 8.0 (https://www.targetscan.org/vert_80/, accessed on 12 October 2021), the most common on-line software predicting the relationship between miRNAs and targets. We identified three genes that met our selection criteria, beclin1, autophagy related (BECN1), autophagy related 14 (ATG14) and autophagy related 13 (AT13) in 409 potential targets (Supplementary Table S1). To further narrow down target candidates, we constructed wild-type (Wt) and mutant-type (Mut) luciferase report systems of BECN1, ATG14 and AT13 mRNA 3′UTR in pmirGLO vector (Supplementary Figure S2b). The reporter assays showed that the ectopic miR-371a-5p expression dramatically suppressed the luciferase activity of Wt BECN1 mRNA 3′UTR in 293T cell and SMMC-7721 cell, while the suppressive effect was abolished in Mut BECN1 mRNA 3′UTR report systems (Figure 3d). The phenomenon was not observed in the ATG13 and ATG14 mRNA 3′UTR report systems (Supplementary Figure S2c,d). Consistent with this, WB results showed that the ectopic miR-371a-5p reduced BECN1 protein expression in HepG2 cell and vice versa in SMMC-7721 cell, while the protein expressions of ATG13 and ATG14 were not altered (Figure 3e). Moreover, we found that the increased effects of the miR-371a-5p silencing on the autophagy-related phenotypes in SMMC-7721 cell could be reversed by silencing BECN-1 (Figure 3b,c and Supplementary Figure S2e,f). We also found that the decreased effects of the miR-371a-5p over-expression on the autophagy-related phenotypes in HepG2 cell could be rescued by the re-expression of BECN1 without 3′UTR (Figure 3b,c and Supplementary Figure S2e,f).

These results indicated that miR-371a-5p regulated HCC cell autophagy by suppressing BECN1, not ATG13 or ATG14 expression.

### 2.4. Inhibition of BECN1-Dependent Autophagy Is Essential for the miR-371a-5p-Caused Sensitivity of HCC Cells to Oxaliplatin In Vitro

To determine whether BECN1 is indispensable to the miR-371a-5p function, we firstly evaluated the effect of BECN1 on the response of HCC cells to OXA. We found that HepG2 cell silencing BECN1 showed a decreased IC50 to OXA and an elevated OXA-induced apoptosis compared to the control (Supplementary Figure S3a,b), while SMMC-7721 cell over-expressing BECN1 showed opposite results (Supplementary Figure S3c,d).

**Figure 4:**
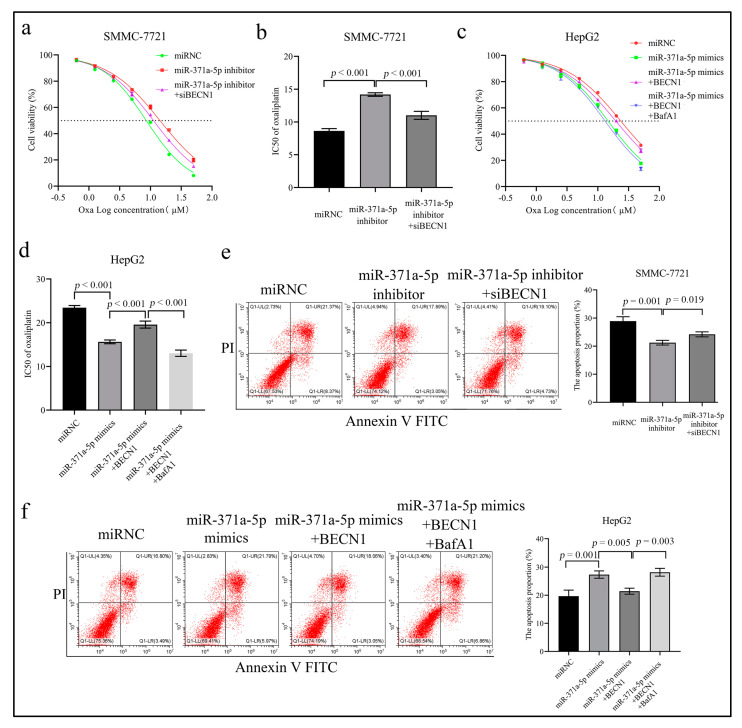
Inhibition of BECN1-dependent autophagy is essential for the miR-371a-5p-caused sensitivity of HCC cells to oxaliplatin. (**a**–**d**) The cell viability graph and the OXA IC50 statistical histogram in SMMC-7721 cell (**a**,**b**) and HepG2 cell (**c**,**d**) following the corresponding treatment. (**e**,**f**) The representative FCM images and statistical histogram of OXA-induced apoptosis in SMMC-7721 cell (**e**) and HepG2 cell (**f**) following the corresponding treatment. Three independent experiments were performed.

We then performed rescue assays to investigate the mediated effect of BECN1-dependent autophagy on the role of miR-371a-5p in the OXA response in vitro. We found that the miR-371a-5p silencing increased the OXA IC50 compared to the control, while the increased effect could be attenuated by the BECN1 silencing in SMCC-7721 cell (Figure 4a,b). We also found that the miR-371a-5p over-expression reduced the OXA IC50 compared to the control, while the reduced effect could be rescued by the re-expression of BECN1 without 3′UTR in HepG2 cell (Figure 4c,d). Importantly, we found the rescued effects of re-expression of BECN1 without 3′UTR on the OXA IC50 reduction by the miR-371a-5p over-expression were abolished following bafilomycin A1 (BafA1) treatment (Figure 4c,d). Consistent with these results, the miR-371a-5p silencing reduced OXA-induced apoptosis compared to the control, while the reduced effect could be rescued by the BECN1 silencing in SMMC-7721 cell (Figure 4e). Inversely, the miR-371a-5p over-expression increased OXA-induced apoptosis compared to the control, while the increased effect could be abolished by the re-expression of BECN1 without 3′UTR in HepG2 cell (Figure 4f). Importantly, the abolished effect of the BECN1 re-expression on the increased apoptosis by miR-371a-5p over-expression was reversed following BafA1 treatment (Figure 4f). These results indicated that miR-371a-5p enhanced the response of HCC cells to OXA by inhibiting BECN1-dependent autophagy.

### 2.5. BECN1 Is Down-Regulated in HCC Tissues and Associated with HCC Malignant Characteristics

**Figure 5:**
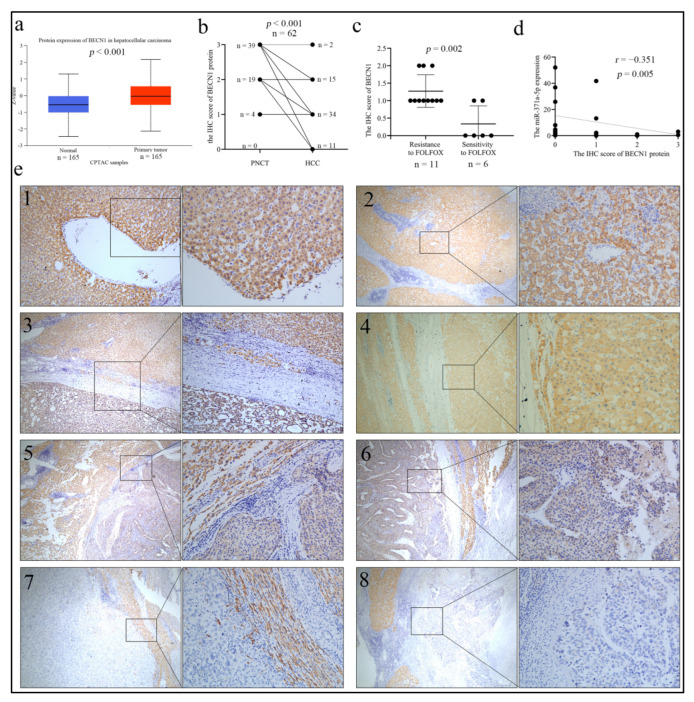
BECN1 is down-regulated and associated with malignant properties in HCC. (**a**) The BECN1 proteomic result from the CPTAC database in 165 normal liver tissues and 165 primary HCC tissues. (**b**,**c**) The IHC staining score of the BECN1 protein in PNCT and HCC tissues (*n* = 62) (**b**), and in HCC tissues from 11 FOLFOX4-resistant and 6 FOLFOX4-sensitive patients (**c**). (**d**) Pearson’s correlation analysis of the IHC staining score of the BECN1 protein with miR-371a-5p expression in 62 HCC tissues. (**e**) The BECN1 protein was detected by IHC and the representative images were shown. (**e1**) Strong positive expression of BECN1 in adjacent normal liver tissues (×100, ×200). (**e2**) Strong positive expression of BECN1 in the cirrhotic liver tissues (×40, ×200). (**e3**) Moderate positive expression of BECN1 in HCC tissues with high differentiation compared with strong positive expression in adjacent liver tissues (×40, ×100). (**e4**) Strong positive expression of BECN1 in HCC tissues with high differentiation and adjacent liver tissues (×40, ×200). (**e5**) Moderate positive expression of BECN1 in HCC tissues with moderate differentiation compared with strong positive expression in adjacent liver tissues (×40, ×200). (**e6**) Weak positive expression of BECN1 in HCC tissues with moderate differentiation compared with strong positive expression in adjacent liver tissues (×40, ×200). (**e7**,**e8**) Negative expression of BECN1 in HCC tissues with low differentiation compared with strong positive expression in adjacent liver tissues (×40, ×200).

Having identified the negative regulatory effect of miR-371a-5p on BECN1 in the response of HCC cells to OXA, we speculated that the BECN1 expression was down-regulated in HCC. Unexpectedly, the data from the CPTAC database, a web resource for analyzing cancer OMICS data, showed that the proteins of BECN1 were dramatically up-regulated in HCC tissue samples compared to in normal tissues (Figure 5a). To verify this, we detected the BECN1 protein expression in the 62 paraffin-embedded tissue samples. Oppositely, the IHC result showed that the BECN1 protein expression was significantly lower in HCC tissues than in adjacent non-cancer liver tissues (Figure 5b,e). We found that the BECN1 expression was higher in tissues from FOLFOX4-resistant HCC patients than in tissue from FOLFOX4-sensitive HCC patients (Figure 5c).

Moreover, Pearson’s correlation analysis showed there was an inverse correlation between the BECN1 protein expression and miR-371a-5p expression in HCC tissues (Figure 5d). We then evaluated the contributions of the BECN1 protein expression to HCC malignant characteristics based on their IHC staining extent. As shown in Figure 5c and Table 2, the BECN1 protein expression was negatively associated with tumor size and differentiation. These results indicated that the BECN1 protein was down-regulated in HCC and negatively associated with HCC malignant characteristics.

## 3. Discussion

Most HCC was diagnosed in advanced stages, when patients had few chemotherapeutic options. The OXA-based FOLFOX4 chemotherapy regimen has recently exhibited active efficacy and excellent safety and been approved for advanced HCC in China [6,22]. However, the development and neglect of resistance to the regimen reduces its clinical benefits. Therefore, it is urgently needed to identify reliable biomarkers associated with the response of patients to the regimen.

In this study, we found that miR-371a-5p expression was higher in HCC tissue and prechemotherapeutic serum from FOLFOX4-sensitive patients than in these from FOLFOX4-resistant patients. Interestingly, there was no significant difference in postchemotherapeutic serum between patients from the two group. One possible explanation for this result is that the FOLFOX4 regimen could suppress miR-371a-5p expression, which was supported by the result that the miR-371a-5p serum level was decreased after patients underwent FOLFOX4 treatment. In fact, we detected the decreased miR-371a-5p expression following OXA treating HCC cells in an ongoing study (data not shown). In addition, we found that the miR-371a-5p serum level was decreased in FOLFOX4-sensitive patients after the chemotherapy, whereas the phenomenon was not observed in FOLFOX4-resistant patients. This finding appears to indicate the possibility that the miR-371a-5p serum level could be a potential biomarker for monitoring the response of HCC patients to FOLFOX4 treatment.

Although miR-371a-5p has been identified to function as an onco-miRNA in HCC [11,12,23], little is known about the role of miR-371a-5p in chemotherapy response [13]. Based on the differential expression of miR-371a-5p between FOLFOX4-sensitive and resistant patients in clinical settings, we speculated that miR-371a-5p may be involved in the regulation of the therapy response. To test it, we explored the effect of miR-371a-5p on the response of HCC cells to OXA, the main component of FOLFOX4 regimen, which exhibited better efficacy and safety than other parallel chemotherapeutic agents and was preferred as a postoperative maintenance therapy in advanced HCC patients [22]. Here, we found that there was a potentially inverse correlation between miR-371a-5p expression and the IC50 of HCC cells to OXA, suggesting that miR-371a-5p exerted a positive role in coordinating the response of HCC cells to OXA. As expected, gain- and loss-of-function assays showed that the miR-371a-5p silencing lowered the response of SMMC-7721 cell to OXA, and the miR-371a-5p over-expression showed an opposite effect in HepG2 cell. These results were consistent with the finding that miR-371a-5p promotes the response of colorectal cancer cells to cetuximab [13]. What puzzles us was the effect of miR-371a-5p on the OXA response was observed in HCC cells with the inhibitor or mimics and OXA co-treatment for 72 h, but not for 48 h. We presumed that it may be required for the pretreatment of HCC cells with the miR-371a-5p inhibitor or mimics in the reduced or enhanced OXA-induced cytotoxicity. To verify this conjecture, we designed miR-371a-5p inhibitor and mimics pretreatment assays (details were shown in the material section). We found that there was no difference in the response of HCC cells to OXA between 72 h miR-371a-5p inhibitor treatment and the miR-371a-5p inhibitor pretreatment, as well as between 72h miR-371a-5p mimics treatment and the miR-371a-5p mimics pretreatment. Hence, we have reason to believe that miR-371a-5p could play an important role in reducing OXA side effects.

To explore the underlying mechanism by which miR-371a-5p enhances the OXA response, we executed bioinformatics analysis for functions of miR-37a-5p target genes. The enrichment of them in the autophagy pathway caught our eyes. Gain- and loss- of- miR-371a-5p assays showed that the miR-371a-5p over-expression repressed autophagy-related phenotypes and vice versa, which was consistent with a most recent study [21]. Nevertheless, whether autophagy is responsible for the effect of miR-371a-5p on the response of HCC cells to OXA still needs to be identified. Bioinformatics analysis screened three candidate mediators related to autophagy. Mechanically, we found that miR-371a-5p suppressed the BECN1 expression by binding to the 3′UTR of BECN1 mRNA. Functionally, rescue assays showed that the BECN1 silencing restored the reduced effect of miR-371a-5p inhibitor on the OXA response, and the non-target BECN1 re-expression reversed the increased effect of miR-371a-5p mimics on the OXA response. Importantly, the autophagy blockage with BafA1 eliminated the effect of the non-target BECN1 re-expression. These results demonstrate that miR-371a-5p enhances the response of HCC cells to OXA by target suppression of BECN1-dependent autophagy.

In sharp contrast with the contribution of miR-371a-5p to the response of HCC cells to OXA, we found that miR-371a-5p expression in HCC tissue, as well as in serum from prechemotherapeutic HCC patients was positively associated with the size and grading of HCC. Additionally, previous studies have demonstrated that miR-371a-5p promotes proliferation of HCC cells by inducing G1/S transition [11]. These findings indicate that miR-371a-5p fulfills a dual function in cancer development and therapy. The duality seems paradoxical, while it is a common phenomenon. It has been reported that the activity of cancer-promoting gene Thymidine Phosphorylase (TP) is necessary for the activation of various chemotherapeutic agents [24]. Similarly, inactivation of tumor suppressor gene p53 strengthens the sensitivity to multiple chemotherapeutic drugs [25]. Moreover, the tumor dormancy theory indicates that conventional chemotherapy preferentially eradicates actively proliferating cancer cells, whereas the relatively quiescent or slowly proliferating ones escape from the therapy and survive by inducing autophagy [26,27], which may provide a decent explanation for the duality.

To our surprise, miR-371a-5p expression data from HCC patients in the two age groups with a cut-off age of 50 reached statistical significance. On the one hand, the difference may be due to the effect of age on the expression of miRNAs. This viewpoint is confirmed by numerous studies [28,29,30], and an earlier study that the age-dependent 1a, 25 dihydroxy vitamin D3 and testosterone decline results in miR-371a-5p down-regulation reinforces it [31]. On the other hand, the impact of age on HCC malignant behaviors could contribute to the difference [32,33]. Additionally, the difference in the association of miR-371a-5p expression with serum AFP level did not display the consistency in tissue level and serum level. In our opinion, it may be due to the limitation of the small sample size, large variety of a clinical setting and the impacts of the FOLFOX4 regimen on miR-371a-5p expression. Further studies with a large sample size and investigating the effects of the component of the FOLFOX4 regimen on the expression of miR-371a-5p are necessary to clarify the issue. It is worth noting that the serum miR-371a-5p level of patients was substantially decreased after HCC resection, which may provide a potential biomarker for assessing the degree of tumor resection. It also seems like a possibility that dynamically monitoring HCC recurrence by detection of miR-371a-5p.

According to the negative regulatory effect of miR-371a-5p on BECN1, the BECN1 expression should be down-regulated in HCC tissue. Unexpectedly, proteomic results from the CPTAC database indicate that the BECN1 protein expression was up-regulated in HCC tissues compared to in normal tissues, which was paralleled by transcriptomics from the TCGA database at the mRNA level (http://ualcan.path.uab.edu/cgi-bin/ualcan-res.pl, accessed on 12 May 2022). In the present study, however, we detected the diminished BECN1 protein expression in HCC tissues compared to in PNCT by IHC. Consistent with our results, multiple studies demonstrate that both protein and mRNA levels of BECN1 are lower in HCC tissues and cells than in the corresponding normal controls [34,35,36]. To our knowledge, the discrepant expression pattern of BECN1 in HCC tissues may be owing to the differences in testing means and study object, including the tumor histological type, stage, number of patients included and patients’ race [24]. A change of perspective, the discrepancies are just according with the duality of autophagy in HCC [17]. Contrary to the contribution of miR-371a-5p to HCC, BECN1 was negatively associated with the size and grading of HCC but hampered the response of HCC cells to OXA. The negative relationship between BECN1 and miR-371a-5p was confirmed by Pearson’s correlation analysis in HCC tissue.

The lack of in vivo experiments is a major deficiency of the present study. Most researchers currently establish the tumor model with subcutaneous injection of cancer cells in nude mice, and then agents are administrated intraperitoneally to inspect their effects on the tumor growth [4,37]. It is undeniable that the model mimics metabolism and circulation of agents in the body to some extent, while in our opinion, the effects of the specific tumor micro-environment on agents and cancer cells are neglected. A spontaneous tumor model from HCC would be required to explore the effect of miR-371a-5p on the response of HCC cells to OXA. This is currently impossible but could be done in future research. Moreover, insufficient follow-up time prevented us from inspecting the effect of miR-371a-5p on the prognosis of HCC patients, so longitudinal records of patients’ survival condition will be a key point for us in next work.

## 4. Material and Methods

### 4.1. Clinical Samples

A total of 62 fresh tissue samples consisting of HCC tissue and the paired adjacent non-cancer liver tissue (PNCT) were obtained from patients undergoing liver resection at Nanchong Central Hospital between January 2019 and January 2021. Among 62 patients, 19 patients underwent preoperative chemotherapy with FOLFOX4 regimen according to the protocol [6]. Briefly, bolus 5-FU 400 mg/m^2^, infusional 5-fluorouracil 600 mg/m^2^ over 22 h on day 1 and 2, bolus leucovorin 100–200 mg/m^2^, and oxaliplatin 85 mg/m^2^, once every 2 weeks. As described in a previous study [4], the appearance of new lesions or the increase of tumor growth > 30% were defined as the FOLFOX4 resistance while the increase of tumor growth < 20% defined as the FOLFOX4 sensitivity following two months of chemotherapy. Based on this, we included 11 FOLFOX4-resistant and 6 FOLFOX4-sensitive patients in this study. Blood samples were collected from the 62 HCC patients on the day before chemotherapy (named as prechemotherapy), the day before operation (named as preoperation or postchemotherapy if patients underwent FOLFOX4 therapy) and on the seventh day after operation (named as postoperation) and 11 healthy volunteers (controls). All the blood samples were allowed to stand for 30 min and then fresh serum was separated by centrifuging at 4 °C, 4000 rpm for 5 min.

All fresh samples were stored at −80 °C until used. The clinicopathological characteristics of all the patients were shown in Table 1. All human experiments were carried out with the approval of the ethics committee of the Nanchong Central Hospital and the written informed consent was obtained from patients.

### 4.2. Cell Lines and Culture

Human HCC cell lines HepG2, SMMC-7721, Huh-7, MHCC-97L and human normal liver cell line LO2 were obtained from Shanghai iCell Bioscience Inc, China. Human embryonal kidney 293T (293T) cell came from the previously frozen cell [38]. All the cell lines were authenticated by short tandem repeat assay. The 293T, Huh-7 and MHCC-97L cells were cultured in DMEM medium (Gibco, Waltham, MA, USA), the SMMC-7721 and LO2 cells in RPMI 1640 medium (Gibco) and the HepG2 cell in MEM medium (Gibco), supplementing with 10% fetal bovine serum (HyClone, Logan, UT, USA) and 1% penicillin/streptomycin (Sigma-Aldrich, St. Louis, MO, USA) at 37 °C in 5% CO_2_.

### 4.3. Vectors, Oligonucleotides and Cell Transfection

Commercial pcDNA3.1-EGFP-BECN1 vectors over-expressing BECN1 and the small interfering RNA (siRNA) of BECN1 (sense, 5′-CUGGACACGAGUUUCAAGATT-3′, antisense, 5′-UCUUGAAACUCGUGUCCAGTT-3′) were purchased from Wuhan GeneCreate Biological Engineering Co., Ltd. (Wuhan, China). Oligonucleotides of miR-371a-5p mimics and the miR-371a-5p inhibitor (5′-TTTTAACATTGCACT-3′) were purchased from Guangzhou Ribobio Co., Ltd. (Guangzhou, China). Lipofectamine 3000 (Invitrogen, Waltham, MA, USA) was used as a carrier to transfect target cells according to the protocol.

### 4.4. Drug Treatment and Grouping

For the detection of the half-maximal inhibitory concentrations (IC50), briefly, HCC cells were treated with OXA at a concentration gradient of 0, 0.625,1.25, 2.5, 5, 10, 20 and 50 μM for 48 or 72 h, and then cell viabilities were detected by Cell Counting Kit-8 (Dojindo, Kumamoto, Japan) method in the Thermo Scientific ™ Varioskan ™ LUX Multimode microplate reader (Thermo Scientific, Waltham, MA, USA) at 490 nm. The software GraphPad Prism 8.0 was used to calculate the IC50. For the analysis of OXA-induced cytotoxicity, HCC cells were treated with 10 μM OXA for 48 or 72 h, and then the FACS Aria cytometer (BD Bioscience, Franklin Lakes, NJ, USA) was used to detect apoptosis using the Annexin V-FITC/PI Apoptosis Assay Kit (Phygene, Fuzhou, China). Bafilomycin A (Sigma-Aldrich) was used to treat HCC cells at a final concentration of 0.2 μM.

In the 48 h or 72 h miR-371a-5p inhibitor/mimics group, HCC cells were co-treated for 48 or 72 h with miR-371a-5p inhibitor/mimics (RiboBio, Guangzhou, China) and OXA (Sigma-Aldrich). In the miR-371a-5p inhibitor/mimics pretreatment group, HCC cells were pretreated for 24 h with miR-371a-5p inhibitor/mimics, then OXA was added to medium and co-treated the cells for 48 h. In rescue assays, HCC cells were co-treated for 72 h with OXA and corresponding agents.

### 4.5. Quantitative Real-Time Polymerase Chain Reaction (QPCR)

Total RNAs in tissues and cells were isolated using the TRIzol method (Invitrogen) according to the manufacturer’s instructions. In the process of serum RNA extraction, 1 ug lyophilized cel-miR-39-3p (Qiagen, Hilden, Germany) was added as exogenous reference according to the provider recommendations and a miRNeasy serum/plasma kit was used to extract serum cell-free miRNAs according to manufacturer’s protocol. The Spectrophotometer ND-1000 (NanoDrop Technologies, Wilmington, NC, USA) was used to detect RNA concentration. The expression levels of miRNAs were determined using All-in-One miRNA qRT-PCR Detection Kit (GeneCopeia, Rockville, MD, USA) in the ABI 7500 System (Applied Biosystems, Foster City, CA, USA) according to the protocol. The relative expression of miR-371a-5p in tissues and cells were normalized to U6 and that in serum to cel-miR-39 using the 2^−ΔΔCt^ method. The primers were as follows:

cel-miR-39, 5′-CAGAGTCACCGGGTGTAAAT-3′;

miR-371a-5p, 5′-TGCGGACTCAAACTGTGGGGGC-3′;

u6, 5′-TGCGGGTGCTCGCTTCGGCAGC-3′.

### 4.6. Western-Blot (WB) and Immunohistochemistry (IHC)

WB and IHC were performed according to established methods in the previous study [38]. Primary antibodies were used at appropriate dilution concentration as follows: WB, anti-ATG13 (Protein Tech, Chicago, IL, USA, 1:2000), anti-ATG14 (Protein Tech, 1:1000), anti-SQSTM1/p62 (Protein Tech, 1:4000), anti-BECN1 (Abcam, Cambridge, UK, 1:1000), anti-LC3B (Protein Tech, 1:2000) and β-actin (Abcam, 1:2500). IHC, anti-BECN1 (Abcam, 1:100).

### 4.7. Luciferase Reporter Assay

As shown in Supplementary Figure S2b, the wild-type (Wt) sequences of ATG13, ATG14 and BECN1 mRNA 3′UTR containing putative miR-371a-5p binding site and their corresponding mutant-type (Mut) sequences were synthesized by Wuhan GeneCreate Biological Engineering Co., Ltd. (China) and, respectively, inserted into the pmirGLO plasmid (Promega, Madison, WI, USA). DNA sequencing was used to validate the eligibility of recombinant plasmid. The recombinant plasmid and miR-371a-5p mimics were co-transfected into 293T and SMMC-7721 cells using Lipofectamine 3000 (Invitrogen). Following 72 h, the Dual-Luciferase Reporter Assay Kit (Promega) was used to determine the luciferase activity according to the manufacturer’s instructions.

### 4.8. Transmission Electron Microscopy (TEM)

Approximately 10^7^ candidate HCC cells were harvested and fixed with 2.5% glutaraldehyde in phosphate buffer for 90 min. The cells were postfixed with 1% osmium tetroxide for 30 min followed by a gradient dehydration using ethanol and acetone and then embedded in Epon 812 resin. The 60-nm ultra-thin sections were obtained using an ultramicrotome (Leica Microsystems, Wetzlar, Germany) and placed on uncoated copper grids. The sections were stained with 3% lead citrate-uranyl acetate and then observed under the TECNAI20 electron microscope (Philips, Amsterdam, The Netherlands). The frequencies of autophagic vacuoles per cell were calculated by averaging the frequencies of autophagic vacuoles in 10 cells.

### 4.9. Statistical Analysis

All statistical analyses were performed using SPSS 22.0 software. Quantitative results with a normal distribution are presented as the mean ± SD and these with an abnormal distribution are presented as median. Based on the result of homogeneity test for variance, Paired student *t* test or Wilcoxon matched-pairs signed rank test was used to analyze statistical significance of the paired data, the independent-samples *t* test or the Mann-Whitney test was used to analyze statistical significance of the data between two groups, one-way ANOVA or the Kruskal-Wallis test was used to analyze statistical significance of the data among groups. Pearson’s correlation analysis was used to determine the correlation. A *p* value < 0.05 was considered as a significant difference.

## 5. Conclusions

In this study, we identify a dual contribution of the miR-371a-5p/BECN1 axis to clinical characteristics and the therapy response in HCC. On the one hand, we find that miR-371a-5p is positively and BECN1 is negatively associated with malignant characteristics of HCC. On the other hand, we reveal that miR-371a-5p is an OXA-sensitive and BECN1 is an OXA-resistant gene. Importantly, we illuminate that miR-371a-5p enhances the OXA response by target suppression of BECN1-dependent autophagy. Reasonable intervention of the miR-371a-5p/BECN1-dependent autophagy pathway will be a promising sensitization strategy in HCC treatment with the OXA-based chemotherapy regimen.

## Figures and Tables

**Table 1:** The relationship between miR-371a-5p expression and clinicopathological characteristics of HCC patients.

Events	*n*	miR-371a-5p in HCC Tissues (Median) ^a^	U/H Value	*p* Value	miR-371a-5p in HCC Serum (Median) ^b^	U/H Value	*p* Value
**Gender**			237	0.434		269	0.842
Male	51	0.356			1.210		
female	11	0.376			0.757		
**Age**			180	0.004		151	0.001
≤50	15	0.934			5.580		
>50	47	0.253			0.616		
**HBsAg**			415	0.561		368	0.208
Negative	24	0.229			0.261		
Positive	38	0.383			1.270		
**Serum AFP (μg/L)**			248	0.001		391	0.221
≤400	33	0.109			0.697		
>400	29	0.797			1.370		
**Liver cirrhosis**			459	0.855		461	0.877
Absence	35	0.356			1.000		
Presence	27	0.376			0.964		
**Tumor size (cm)**			239	0.023		122	< 0.001
≤5.0	45	0.347			0.616		
>5.0	17	0.455			5.580		
**Tumor number**			338	0.820		256	0.115
Single	47	0.361			0.723		
Multiple	15	0.356			2.790		
**Tumor differentiation (grading)**			9.45	0.009		13.7	0.001
Well (1)	11	0.347			0.174		
Moderate (2)	30	0.117			0.246		
Poor (3)	21	1.070 *			2.79 # *		
**Microvascular** **invasion**			374	0.607		322	0.191
Yes	19	0.356			2.600		
No	43	0.376			0.838		

**^a^** miR-371a-5p expression was normalized to that of HCC tissue from a 49-year-old male patient. **^b^** miR-371a-5p expression was normalized to that of the prechemotherapeutic serum from a 49-year-old male HCC patient. # Compared to the tumor with well differentiation, the difference was statistically significant. * Compared to the tumor with moderate differentiation, the difference was statistically significant.

**Table 2:** The relationship between the BECN1 protein expression and clinicopathological characteristics of HCC patients.

Events	*n*	HCC BECN1 IHC Scores (Mean ± SD)	F/t Value	*p* Value
**PNCT**	62	2.560 ± 0.617	12.30	<0.001
**HCC**		1.100 ± 0.762		
**Gender**			1.100	0.278
Male	51	1.180 ± 0.740		
female	11	0.909 ± 0.701		
**Age**			0.026	0.978
≤50	15	1.130 ± 0.915		
>50	47	1.130 ± 0.679		
**HBsAg**			0.386	0.701
Negative	24	1.080 ± 0.654		
Positive	38	1.160 ± 0.789		
**Serum AFP (μg/L)**			0.949	0.347
≤400	33	1.210 ± 0.650		
>400	29	1.030 ± 0.823		
**Liver cirrhosis**			0.514	0.609
Absence	35	1.170 ± 0.747		
Presence	27	1.070 ± 0.730		
**Tumor size (cm)**			2.060	0.043
≤5.0	45	1.240 ± 0.743		
>5.0	17	0.824 ± 0.636		
**Tumor number**			0.026	0.980
Single	47	1.130 ± 0.769		
Multiple	15	1.130 ± 0.640		
**Tumor differentiation (grading)**			29.10	<0.001
Well (1)	11	2.000 ± 0.632		
Moderate (2)	30	1.230 ± 0.430		
Poor (3)	21	0.524 ± 0.602 # *		
**Microvascular invasion**			0.541	0.591
Yes	19	1.050 ± 0.705		
No	43	1.160 ± 0.754		

# Compared to the tumor with well differentiation, the difference was statistically significant. * Compared to the tumor with moderate differentiation, the difference was statistically significant.

## Data Availability

All the datasets supporting our findings are shown in this article.

## Supplementary Materials

The following supporting information can be downloaded at: https://www.mdpi.com/article/10.3390/life12101651/s1, Supplementary Figure S1. There was no significant effect of miR-371a-5p on apoptosis of HCC cells; Supplementary Figure S2. MiR-371a-5p regulates HCC cell autophagy by BECN1-dependent pathway in vitro; Supplementary Figure S3 BECN1 attenuates the response of HCC cells to oxaliplatin and Supplementary Table S1. miR-371a-5p target genes predicted by targetscan 8.0.
