# Supplementary material for: MiR-371a-5p Positively Associates with Hepatocellular Carcinoma Malignancy but Sensitizes Cancer Cells to Oxaliplatin by Suppressing BECN1-Dependent Autophagy
Source: Life (Basel). 2022 Oct 20;12(10):1651. doi: 10.3390/life12101651 (PMC9604925; doi:10.3390/life12101651)
Supplement: Supplementary file 1 [file life-12-01651-s001.zip › Supplementary Figures and legends.pdf]

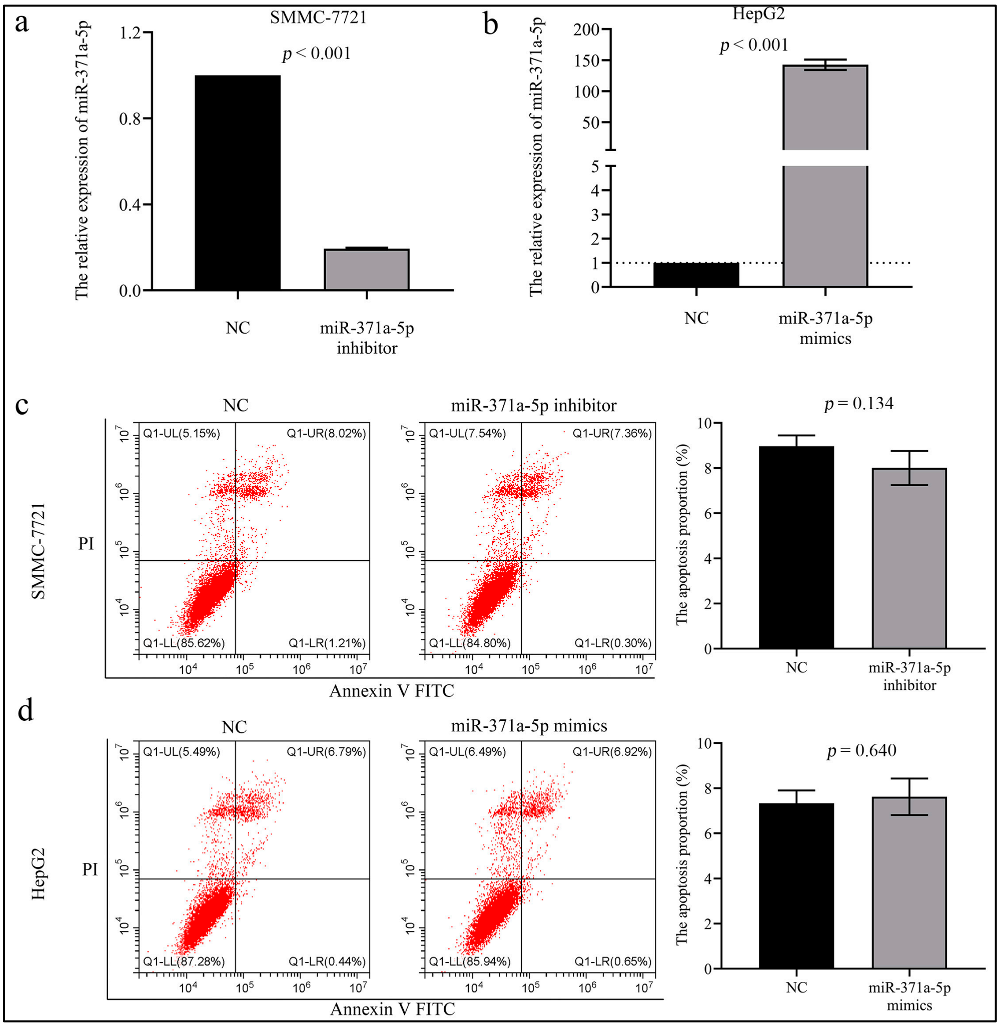

Supplementary Figure S1. There was no significant effect of miR−371a−5p on apoptosis of HCC cells. (a,b) The transfection efficiencies of miR−371a−5p inhibitor (a) and mimics (b) were detected by QPCR. (c,d) The effects of miR−371a−5p inhibitor (c) and mimics (d) on the apoptosis of HCC cells were detected by FCM and the representative images were shown. Three independent experiments were performed.

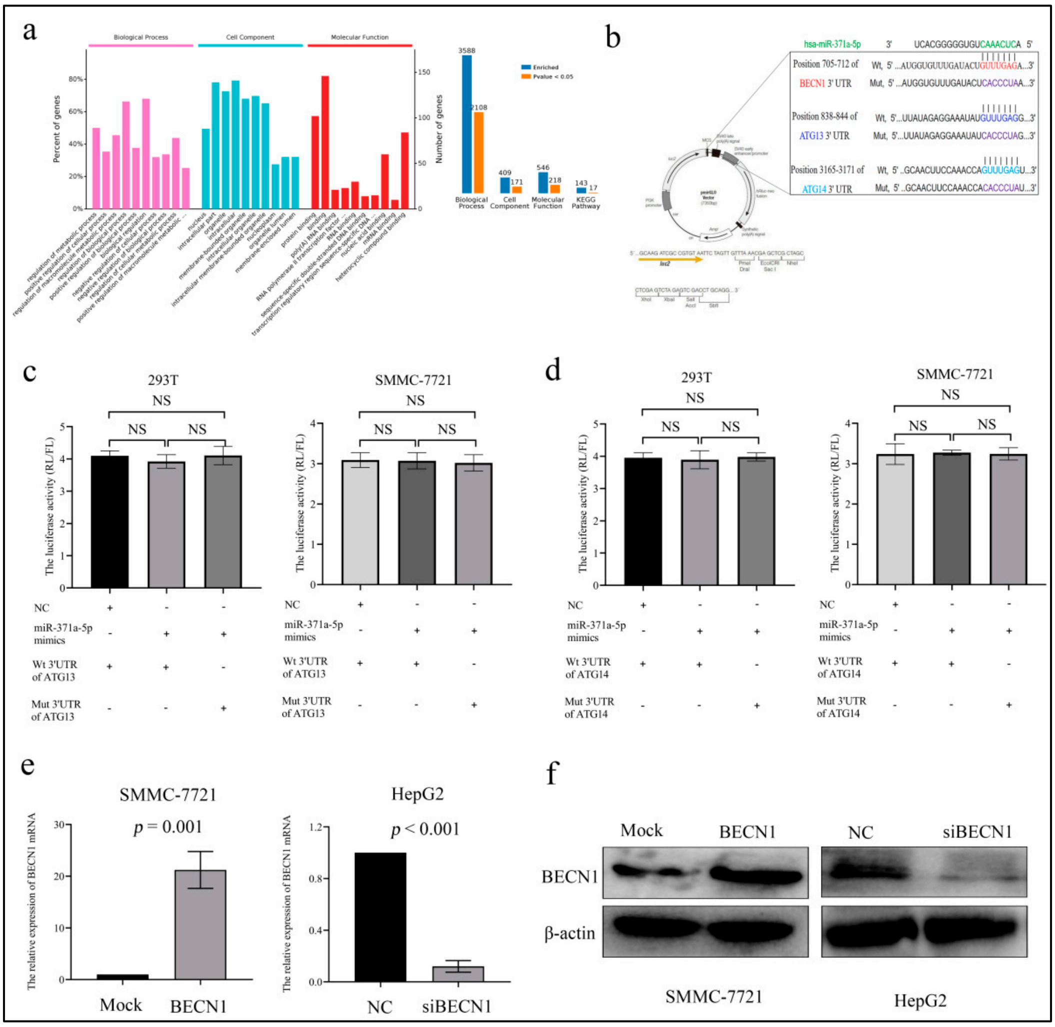

Supplementary Figure S2. MiR−371a−5p regulates HCC cell autophagy by BECN1−dependent pathway in vitro. (a) The analysis fo functions of miR−371a−5p target genes by OMICSBEAN. (b) Predictive binding sites of miR−371a−5p to 3'UTR of BECN1, ATG13 and ATG14 mRNAs by TargetScanHuuman 8.0 and and mutant sites. (c,d) The luciferase activities of Wt and Mut pmirGLO−3'UTRs of ATG13 (c) and ATG14 mRNA (d) in 293T and SMMC−7721 cells following miR−371a−5p mimics treatment (Wt wild−type, Mut mutant−type, NS no significance, RL Ranilla luciferase, FL Firefly luciferase). (e,f) The transfection efficiencies of the BECN1−overexpressing plasmid and siBECN1 were detected by QPCR (e) and WB (f).

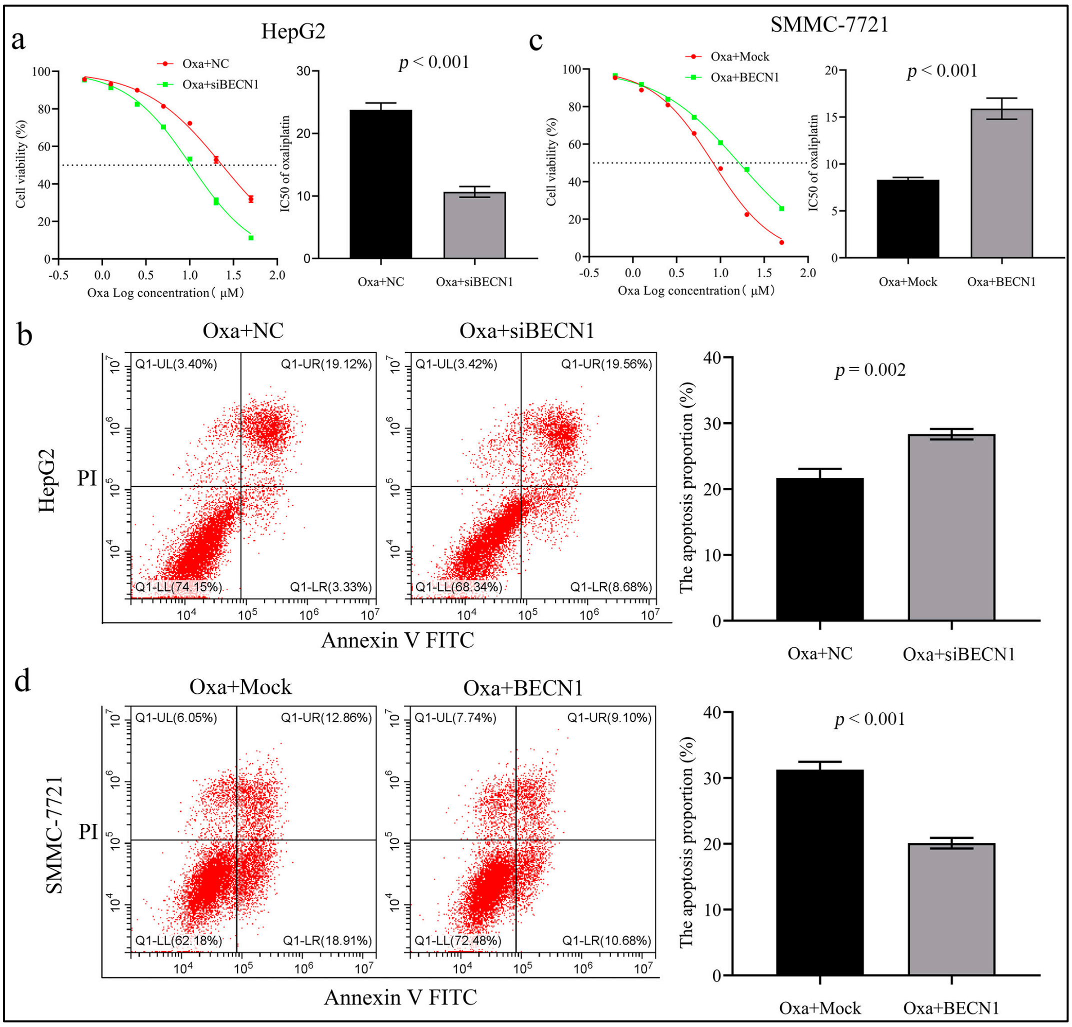

Supplementary Figure S3. BECN1 attenuates the response of HCC cells to oxaliplatin. (a,b) The cell viability graph and the OXA IC50 statistical histogram in HepG2 cell and SMMC−7721 cell following the BECN1 silencing (a) and the BECN1 over−expression (b). (c,d) The representative FCM images and statistical histogram of OXA−induced apoptosis in HepG2 cell and SMMC−7721 cell following the BECN1 silencing (c) and the BECN1 over−expression (d). Three independent experiments were performed.
